# Probing Cell‐Surface Interactions in Fungal Cell Walls by High‐Resolution ^1^H‐Detected Solid‐State NMR Spectroscopy

**DOI:** 10.1002/chem.202202616

**Published:** 2022-11-10

**Authors:** Adil Safeer, Fleur Kleijburg, Salima Bahri, David Beriashvili, Edwin J. A. Veldhuizen, Jacq van Neer, Martin Tegelaar, Hans de Cock, Han A. B. Wösten, Marc Baldus

**Affiliations:** ^1^ NMR Spectroscopy Bijvoet Center for Biomolecular Research Utrecht University Padualaan 8 3584 CH Utrecht (The Netherlands; ^2^ Microbiology Department of Biology Utrecht University Padualaan 8 3584 CH Utrecht (The Netherlands; ^3^ Division of Infectious Diseases and Immunology Department of Biomolecular Health Sciences Utrecht University Yalelaan 1 3584 CL Utrecht (The Netherlands

**Keywords:** cell wall, NMR spectroscopy, peptide, proton detection, schizophyllum commune

## Abstract

Solid‐state NMR (ssNMR) spectroscopy facilitates the non‐destructive characterization of structurally heterogeneous biomolecules in their native setting, for example, comprising proteins, lipids and polysaccharides. Here we demonstrate the utility of high and ultra‐high field ^1^H‐detected fast MAS ssNMR spectroscopy, which exhibits increased sensitivity and spectral resolution, to further elucidate the atomic‐level composition and structural arrangement of the cell wall of *Schizophyllum commune*, a mushroom‐forming fungus from the Basidiomycota phylum. These advancements allowed us to reveal that Cu(II) ions and the antifungal peptide Cathelicidin‐2 mainly bind to cell wall proteins at low concentrations while glucans are targeted at high metal ion concentrations. In addition, our data suggest the presence of polysaccharides containing N‐acetyl galactosamine (GalNAc) and proteins, including the hydrophobin proteins SC3, shedding more light on the molecular make‐up of cells wall as well as the positioning of the polypeptide layer. Obtaining such information may be of critical relevance for future research into fungi in material science and biomedical contexts.

## Graphical abstract

**How** ^**1**^ **H‐detected fast MAS ssNMR spectroscopy** can be applied for in‐depth characterization of the *Schizophyllum commune* cell wall architecture and composition, as well as the elucidation of cell surface‐level interactions. The obtained results reveal the relative abundance and arrangement of the polysaccharide and polypeptide layers and their significance in the binding of ligands to the fungal cell wall.

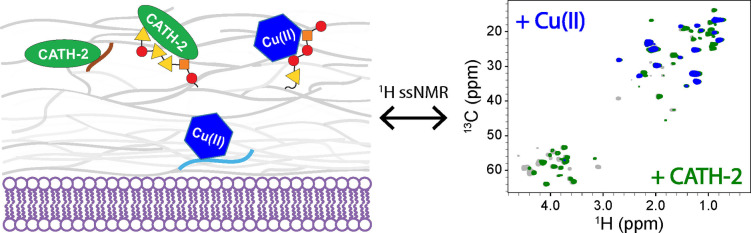

## Introduction

The fungal kingdom is diverse with a predicted number of 2.2 to 3.8 million species.^[1]^ These species are essential for nutrient recycling in nature and for establishing pathogenic or symbiotic interactions with plants, animals and microbes. Fungi also play an important role for human society in the industrial production of enzymes, small molecule compounds such as antibiotics, organic acids,^[2]^ and ultimately in the production of food and feed.^[3]^ In the future, filamentous fungi are likely to play an important role in the transition to a circular economy by their use in bioremediation^[4]^ and in the production of sustainable materials that can replace plastics.^[2]^

The fungal cell wall plays a key role in the functioning of fungi in nature and in biotechnology. It provides mechanical strength and represents a critical barrier for diffusion of molecules,^[5]^ which is of key importance for the use of fungi as cell factories and the production of sustainable materials.^[2]^ The cell wall is also in direct contact with the (a)biotic environment and thereby a prime target for the immune systems of plants and animals to prevent infections.^[6]^ These immune‐stimulatory cell wall molecules are also of interest for use in the clinic. Finally, cell walls contain other bioactive molecules that can bind metals and are therefore important for bioremediation.^[7]^

Despite their importance, relatively little is known about the molecular architecture of fungal cell walls. It is known that the composition of the cell walls varies between species, strains, environmental conditions, and developmental stage.^[8]^ Traditionally, cell wall analysis was based on destructive methods using enzymatic and/or chemical treatments. In the last decade, solid‐state Nuclear Magnetic Resonance (ssNMR) spectroscopy has become a powerful means to analyze whole cells and cell walls as well as cell surface level interactions (see, for example, Ref. [9]). These ssNMR studies provided a paradigm shift in our atomistic view of the fungal cell wall.[^9d^, ^10^] For instance, ^13^C‐detected magic angle spinning (MAS) ssNMR spectroscopy showed^[10d]^ that the cell wall of the mushroom forming fungus *Schizophyllum commune* contains a rigid core that consists of α‐(1,3)‐glucan, β‐(1,3)(1,6)‐glucan, branched mannose, fucan, and β‐(1,4)‐chitin and a mobile exterior comprising β‐(1,3)(1,6)‐glucan, mannan and α‐glucan residues as well as amino acids.

The use of multidimensional ssNMR spectroscopy with ^1^H evolution^[11]^ or detection^[12]^ periods has already been demonstrated in plant and bacterial cell walls, respectively. Here we show the use of ^1^H‐detected MAS ssNMR spectroscopy at high and ultra‐high magnetic field to reduce spectral overlap. The increased sensitivity and resolution allowed us to refine our previous model of the *S. commune* cell wall organization and to study how ligands i. e., metals and antifungal peptides, interact with the fungal cell wall surface at atomic resolution.

## Results and Discussion

**Figure 1:**
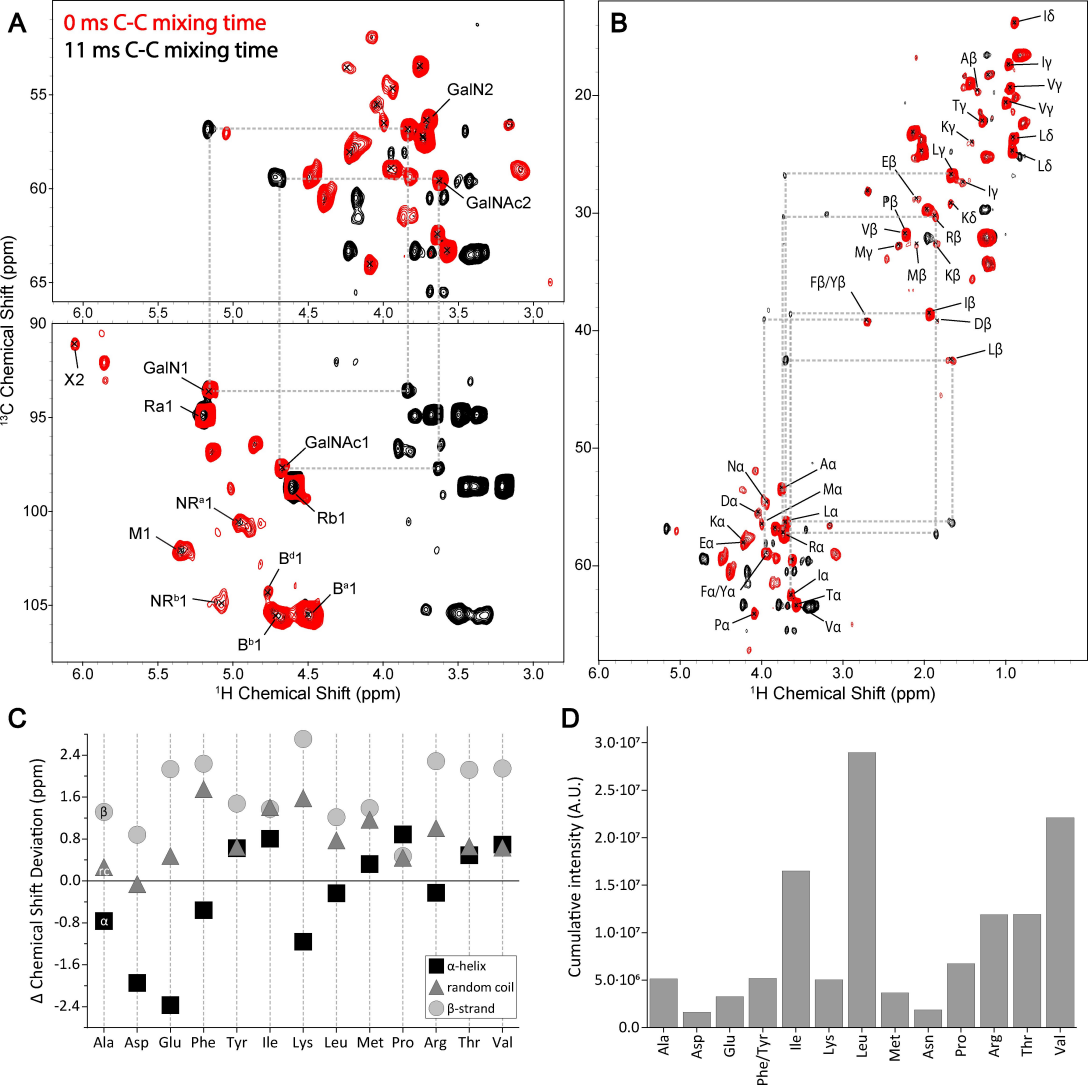
Scalar‐based hCcH ssNMR spectroscopy experiments of the *S. commune* cell wall. Cut‐outs from scalar 2D ^1^H‐detected CH correlation spectra displaying the amino‐acid Cα and polysaccharide C1 regions (**A**) and the amino acid backbone and sidechain regions (**B**) with 0 ms (red) and (black) 11 ms of C−C mixing. Dashed lines (grey) highlight the appearance of cross‐peaks. (**C**) Δ Chemical Shift deviation of the cell wall amino acid chemical shifts from isotropic random coil, α‐helix and β‐strand secondary structure chemical shift. (**D**) Cumulative intensity of cell wall amino acids. Note that Cys was not analyzed in our data. See Supporting Information for further information.

^1^H‐detected ssNMR spectroscopy was applied for in‐depth characterization of uniformly (^13^C,^15^N)‐labeled *S. commune* cell wall material. ^13^C resonance assignments were based on chemical shifts previously^[10d]^ retrieved from 2D ^13^C−^13^C correlation spectra of *S. commune*. De novo ^1^H assignments were obtained from the (^1^H,^13^C) correlation spectra by assuming only one‐bond correlations to appear for each molecular species under the experimental ssNMR conditions. The resulting values compared favorably to database values^[13]^ and were, in addition, validated using previous reports (See Supporting Information, Tables S3–S5 and cited references). We employed tailored scalar‐based hC(c)H ^1^H‐detection experiments to probe mobile compounds of the cell wall for further assignment of flexible cell wall species (Figures 1A and S1). These experiments confirmed the presence of polysaccharides containing N‐acetyl galactosamine (GalNAc) and its deacetylated variant (GalN), which was not previously^[10d]^ reported for *Schizophyllum commune*, but has been described in fungal systems via ^13^C‐detected ssNMR spectroscopy.^[10a]^ Furthermore, C−C mixing facilitated a near full assignment of the amino acids present in the dynamic domain of the cell wall (Figure 1B, see dashed lines). The resonance frequencies indicate that these amino acids are mostly unstructured, thus occurring in random coil conformation, while Glu, Lys, Leu, and Asn exhibit a slight tendency towards α‐helical Cα chemical shifts^[14]^ under the conditions used for cell wall isolation (Figure 1C, Supporting Information). Notably, we found strong NMR intensities for hydrophobic amino acids (Ile, Leu and Val) and also detected threonine (Figure 1D), which in line with the presence of SC3 (Figure S2).^[15]^ This hydrophobin has previously been shown to affect *S. commune* cell wall architecture^[16]^ and can interact with β‐glucans.^[17]^ Lastly, we were able to probe the presence of NMR signals stemming from dynamic lipid chains (Vide infra, Figure 3A) likely belonging to the *S. commune* cell membrane in our preparations by applying scalar‐based experiments, which utilize *J*‐couplings between chemically bonded. nuclear spins to select for mobile molecular species^[18]^.

**Figure 2:**
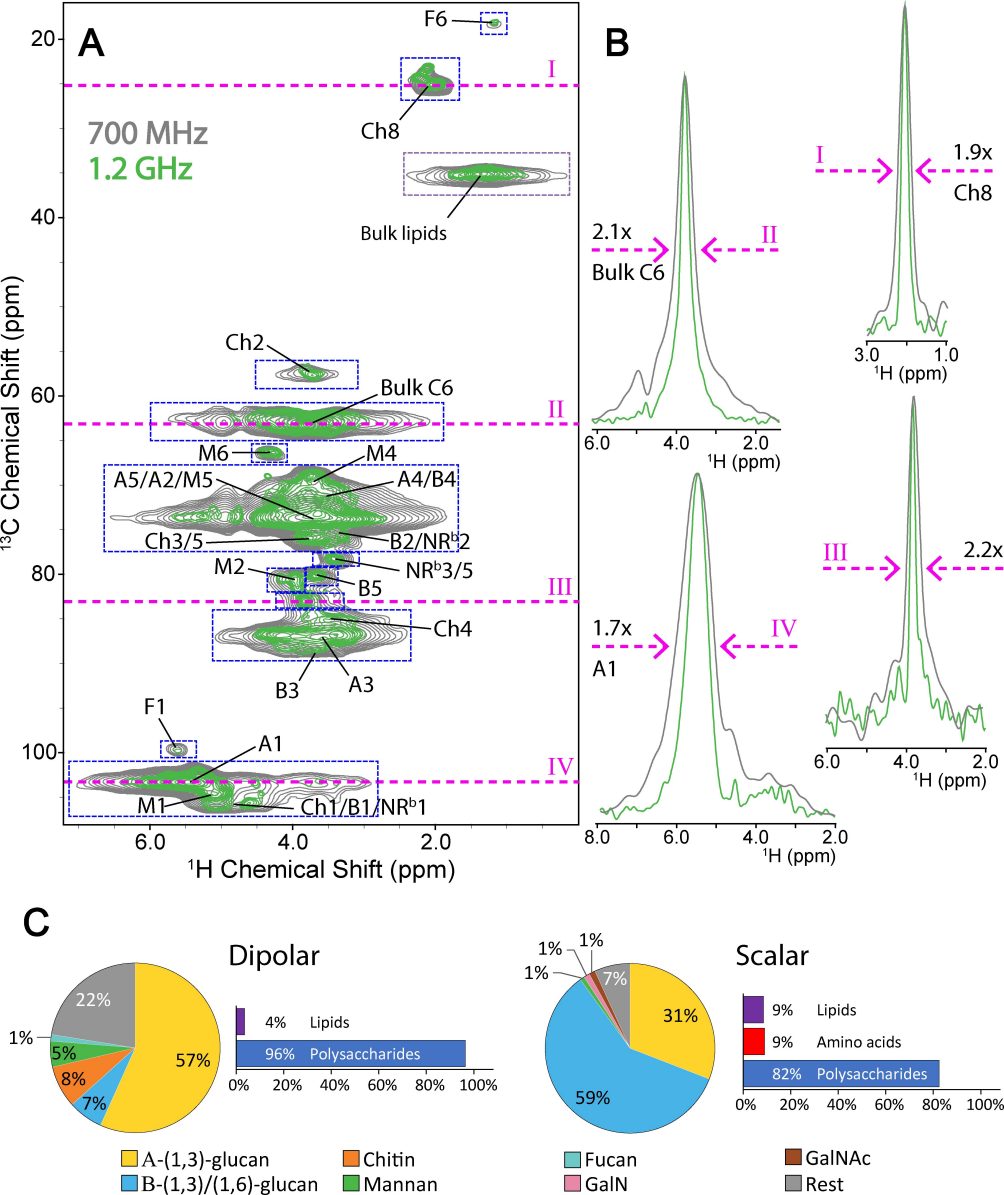
Peak linewidth improvement at 1.2 GHz vs. 700 MHz and the composition of the *S. commune* cell wall. (**A**) An overlay of dipolar 2D CH‐correlation spectra recorded at 1.2 GHz (green) and 700 MHz (grey), scaled to noise level. (**B**) 1D slices (magenta lines) from the 2D spectra demonstrate improvement in peak linewidth at half height, scaled to maximum peak height. (**C**) The relative abundance of rigid (dipolar) and flexible (scalar) cell wall polysaccharide species derived from 2D CH‐correlation spectra. The employed integration areas for the quantification of rigid species are denoted by dashed boxes (blue) in (A). The legend for both pie charts is shown at the bottom, with GalN and GalNAc denoting galactosamine‐containing polysaccharides (pink), and N‐acetyl galactosamine‐containing polysaccharides (brown), respectively.

Dipolar‐based ^1^H‐detection experiments were employed to probe rigid cell wall components, mainly polysaccharides. Due to the manifold of conformations that rigid polysaccharide chains can adopt in the cell wall, peaks belonging to polysaccharides forming the rigid cell wall core exhibited increased linewidth ranging from 3.0 to 4.0 ppm (at 700 MHz, Figure 2A, grey) in the ^1^H dimension, whereas correlations belonging to the flexible sugar moieties appeared well‐resolved and dispersed. Interestingly, analogous dipolar‐based 2D ^1^H‐^13^C experiments conducted at 1.2 GHz (Figure 2A, green), showed a remarkable reduction in ^1^H linewidth (Figure 2B) suggesting that the residual linewidth is, at least in part, related to homogeneous broadening effects that are reduced at ultra‐high field.^[19]^ On average, ^1^H linewidths were reduced by a factor of ∼2, (see Table S1) which is in accordance with earlier findings using in vitro preparation^[20]^ and demonstrates the beneficial effect of ultra‐high field NMR instrumentation for in situ studies.

The spectral improvements using ^1^H‐detection allowed us to estimate the relative contribution of various polysaccharide species to the *S. commune* cell wall (see Supporting Information). For this purpose, we integrated broad NMR signals in dipolar‐based 2D spectra (Figure 2A), and found that glucose polysaccharides, predominantly α‐glucans are the major component (Figure 2C, 63 %) of the rigid part of the cell wall. The lower abundance of chitin (8 %) mannan (5 %) and fucan (1 %) corroborates previous HPLC data^[10d]^ and is in agreement with an analysis of ^13^C‐detected double‐quantum single‐quantum 2D experiments that display single‐bond correlations^[21]^ as described by Chakraborty et al.^[10a]^ (Figure S3). The remaining rest value (Figure 2C, grey) could be attributed to undetermined polysaccharide species. To determine the relative contribution of specific polysaccharides to the flexible part of cell wall, we utilized peak heights instead of peak volumes due to the narrower ^1^H linewidth (Figure 1A). In addition, we also determined the ^1^H linewidth for the relevant polysaccharide protons which are of comparable size (Table S12). In a simplified analysis that only includes the measured peak heights, we find that the mobile cell wall is even more dominated by glucose polysaccharides, compared to the rigid cell wall, (Figure 2C, 90 %). The largest contributors to the dominant mobile β‐glucan species were the reducing end β‐(1,3)‐glucans (Figure S4). This strongly hints at the presence of short β‐glucan chains in the flexible cell wall alongside the expected longer β‐(1,3)(1,6)‐glucan chains described previously^[10d]^ (see Figure S5 for molecular structures).

**Figure 3:**
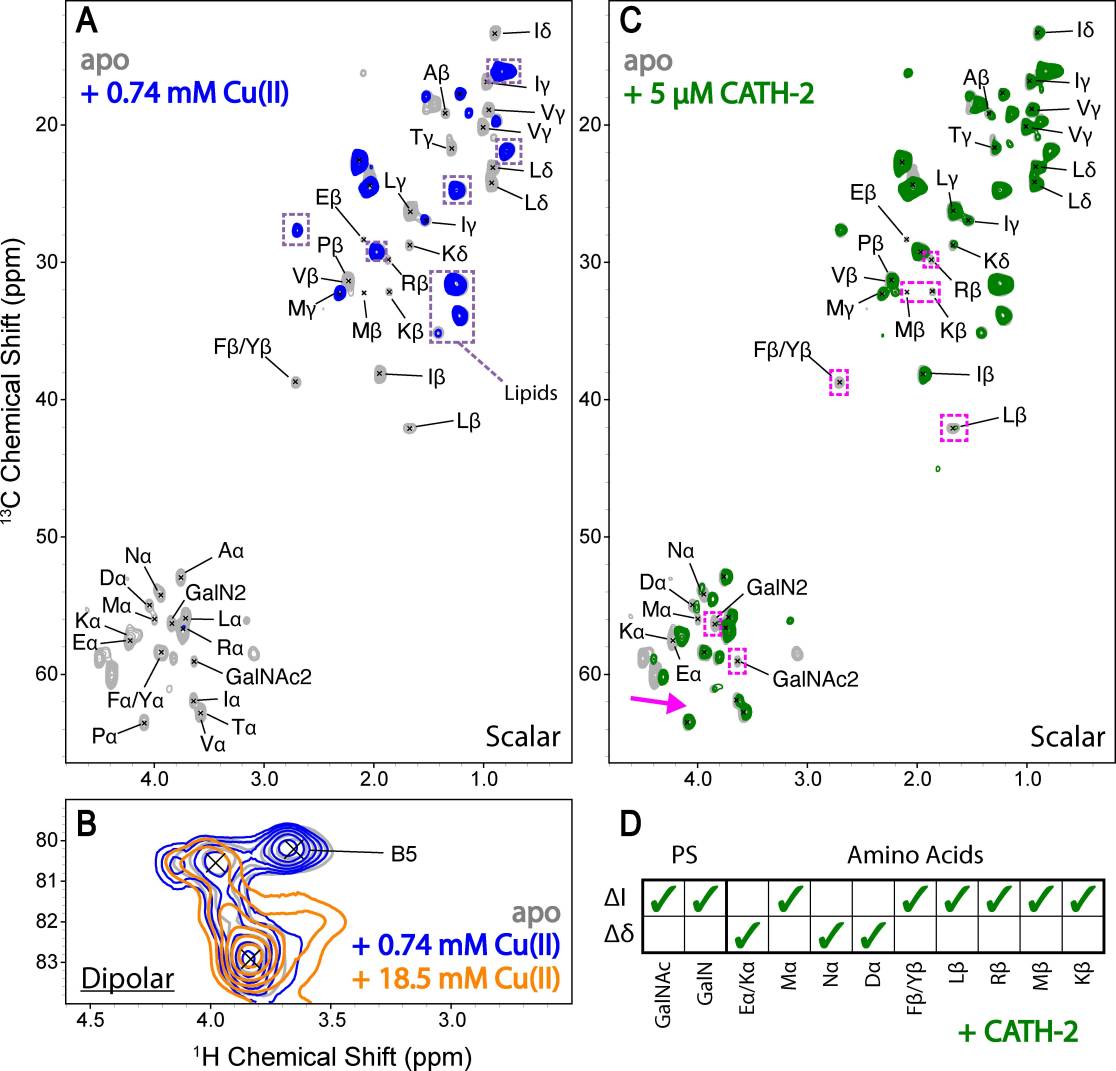
Binding of ligands to the *S. commune* cell wall. (**A**) A cut‐out from an overlay of scalar 2D ^1^H‐detected CH correlation spectra of the amino acid backbone and sidechain regions before (grey) and after incubation with 0.74 mM Cu(II) (blue). Potential lipid resonances are encircled in dashed boxes (purple). (**B**) A cut‐out of dipolar 2D CH correlation spectra before (grey) and after incubation with 0.74 mM Cu(II) (blue) and 18.5 mM Cu(II) (orange). (**C**) A cut‐out from an overlay of scalar 2D ^1^H‐detected CH correlation spectra of the amino acid backbone and sidechain regions before (grey) and after incubation with 5 μM CATH‐2 (green). Major binding effects are denoted with dashed boxes (magenta) in (B) and (C). (**D**) Polysaccharide (PS) and amino acid resonances that undergo changes in signal intensity (Δ*I*) and CSPs (Δ*δ*) upon the addition of CATH‐2.

In a material sciences context, our ^1^H‐detected ssNMR spectroscopy approach represents a powerful tool to reveal the potential binding sites of metal ions to the bioactive molecules in *S. commune* cell surface. To this end, *S. commune* cell wall material was incubated with a 0.74 mM Cu(II) solution and subsequently washed with demineralised water prior to the acquisition of ^1^H‐detected spectra at 700 MHz. Cut‐outs of the resulting ^1^H‐^13^C correlation spectra are shown in Figure 3. The scalar‐based experiments showed that amino‐acid signals readily disappeared upon the addition of metal ions due to the inherent paramagnetic signal quenching effects of Cu(II) (see, for example Ref. [9 f]), while bulk sugar and lipid signals remained less affected (Figures 3A and S6). Notably, our data suggest that additional binding occurs at higher Cu(II) concentrations (Figures S6 and S7) and that ions can then penetrate to the rigid cell wall as evidenced by the disappearance of β‐(1,3)(1,6)‐glucan C5 signal in the dipolar‐based experiments (Figure 3B). At this concentration cell wall material is visibly stained (Figure S8). Taken together, these results reveal that Cu(II) ions mainly bind cell wall proteins at low concentrations and glucans are only targeted at higher concentrations or when all protein binding sites are occupied. The outcome of our ion binding study can be used to elucidate potential molecular targets in the cell walls of other fungal species that may be capable of binding metals more efficiently.

The application of ^1^H‐detected ssNMR spectroscopy in characterizing the binding of antibiotic and antifungal peptides has been a current research focus for medical applications. Previously, we demonstrated antifungal activity of Cathelicidin‐2 (CATH‐2) and cathelicidin‐inspired antimicrobial peptides termed ‘PepBiotics’ against a large set of medically relevant fungi.^[22]^ Here, we employed ^1^H‐detected ssNMR spectroscopy to probe potential binding sites of the antifungal peptide CATH‐2 which can inhibit *S. commune* growth (Figure S9). Using low micromolar concentrations we observed Chemical Shift Perturbations (CSPs) in the Cα amino‐acid region (Figures 3C and S10) and the disappearance of charged (E, D, R, K, Y) amino acid side chain resonances (summarized in Figure 3D) indicating binding to the cell wall proteins. Furthermore, specific polysaccharide, GalN/GalNAc1 and GalN/GalNAc2, signals disappeared and CSPs were observed in the bulk polysaccharide region (Figure S11). Note that most of the CSPs were also seen in the ^1^H spectral dimension (Figure S10), underlining the critical need of ^1^H‐detected ssNMR spectroscopy for the current study. Our results suggest that inhibition of *S. commune* growth by CATH‐2 proceeds at least in part by distinct interactions between the peptide land specific cell wall components (proteins but also polysaccharides). Notably, galactoaminogalactan (GAG) of *Aspergillus fumigatus* shares GalNAc and GalN structures and plays multiple roles in fungal pathogenesis and immune evasion.^[23]^ The described interactions could mediate cell wall changes as was shown previously for the human cathelicidin LL‐37^[24]^ and/or they could induce a mechanism to hinder entry to fungal cell membranes which have been proposed targets for CATH‐2.^[25]^

## Conclusion

**Figure 4:**
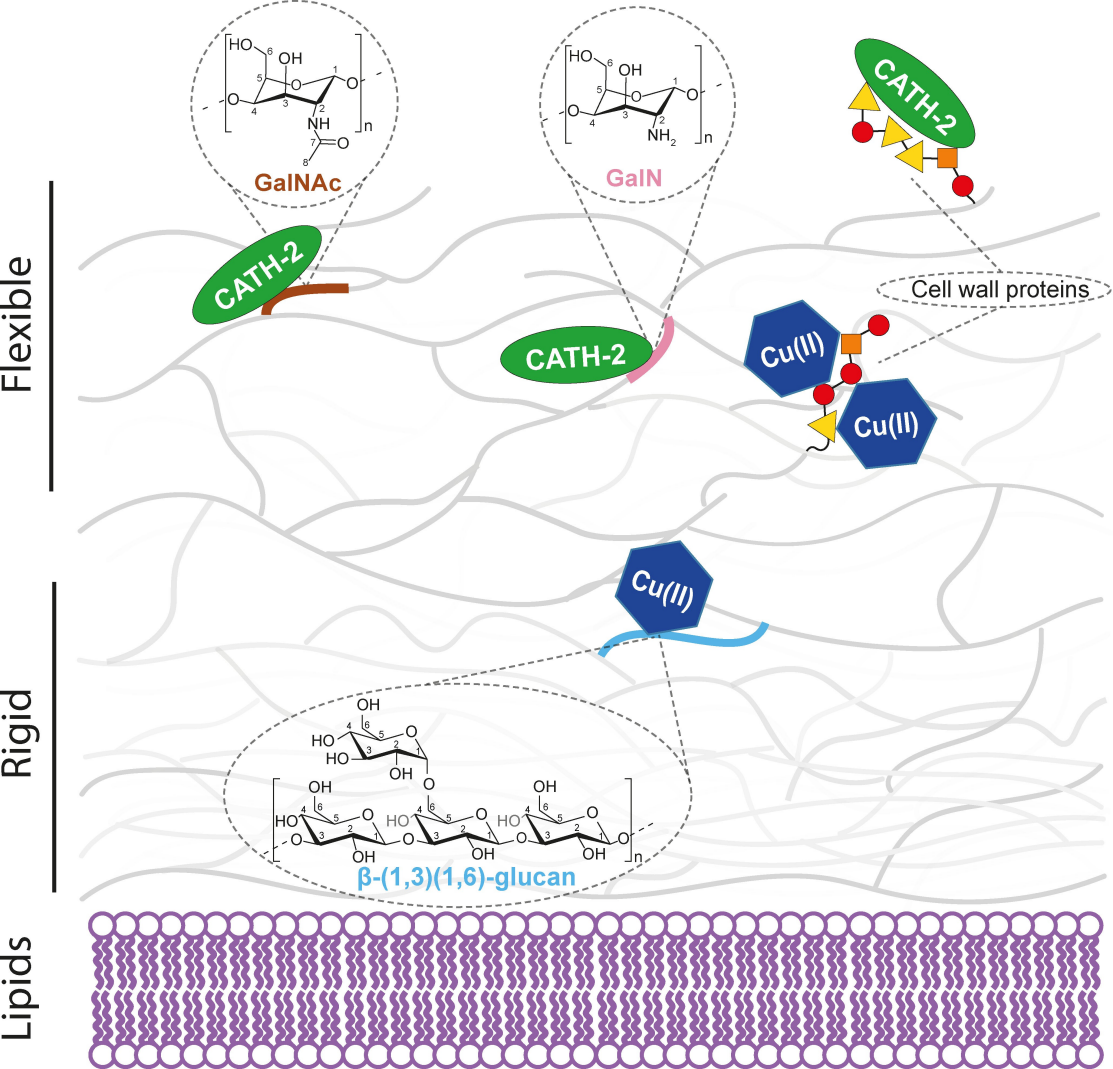
Refined model of the *S. commune* cell wall and its interaction with ions and an antifungal peptide. Cu(II) ion and CATH‐2 binding hotspots identified using ^1^H‐detected ssNMR spectroscopy are denoted in the model. Charged amino acid residues in the peptide chains are denoted by triangles (yellow), while nonpolar uncharged residues are shown as circles (red) and nonpolar aliphatic residues as squares (orange). The model has been adapted from Ehren et al.^[10d]^

We have demonstrated the power of high and ultra‐high field ^1^H‐detected ssNMR spectroscopy to advance our current understanding of the fungal cell wall architecture and its interaction with external binding partners. Our experiments revealed new polysaccharide species and provided structural insight into the proteins present in the *S. commune* cell wall. In particular our data suggest that the cell wall proteins are mostly unstructured and that SC3 is prominently present. Furthermore, we were able to study various polysaccharides and the location in the dynamic and/or rigid portion of the cell wall (Figure 4) in high spectral resolution. In addition, we could resolve atom‐specific binding events in *S. commune* cell walls for positively charged ions and an antifungal peptide. For the cationic CATH‐2 peptide, interactions seem to mainly involve charged amino acids (Figure 4). Interestingly, both type of molecules share a common binding interface, i. e., the polypeptide layer of the cell wall. Next to our ability to determine binding locations of metal ions with complex cell surfaces at the atomic scale, these results also allowed us to refine our current model of the *S. commune* cell wall (Figure 4). Accordingly, amino acids are located close to the outer part of the mobile cell wall, are largely unstructured and undergo strong signal attenuation in the presence of Cu(II) ions. In contrast, bulk polysaccharides and the cell membrane lipids are buried deeper in the *S. commune* cell wall and experience less signal attenuation (Figure 4).

These advancements will allow us to study the binding of various other substrates to intact fungal cell walls, expanding the use of ssNMR‐guided high‐resolution studies on fungi in a broad range of applications from biomaterial and foods science to antifungal research. Importantly, analogous ^1^H‐^13^C correlation experiments could also be performed on unlabelled materials, expanding the use of high‐resolution ^1^H‐detected ssNMR spectroscopy to native settings and processes, including the monitoring of fungal systems under changing environmental conditions.

## Experimental Section

**Culture conditions**: *S. commune* strain H4‐8 A (matA43 matB41; FGSC no. 9210)^[26]^ was grown at 30 °C on *Schizophyllum* minimal medium agar (SCMMA; containing 22 g L^−1^ glucose monohydrate (Sigma‐Aldrich, St Louis, MO, USA), 1.5 g L^−1^ asparagine monohydrate (Sigma‐Aldrich, St Louis, MO, USA), 1 g K_2_HPO_4_ (Merck, Rahway, New Jersey, USA), 0.5 g MgSO_4_ ⋅ 7H_2_O (Sigma‐Aldrich, St Louis, MO, USA), 0.46 g KH_2_PO_4_ (Merck, Rahway, New Jersey, USA), 0.12 mg thiamine‐HCl (Sigma‐Aldrich, St Louis, MO, USA), 5 mg FeCl_3_, trace elements according to Whitaker^[27]^ and 15 g agar (VWR, Radnor, Pennsylvania, USA). SCMMA was inoculated with a 10×10 mm square of mycelium from a colony that had been grown on SCMMA and that had been stored at ‐80 °C. After 6 days, 10 1×1 mm plugs were taken from the periphery of the colony and incubated at 30 °C and 50 rpm in a 50 mL Falcon tube (Greiner Bio‐One, Alphen aan den Rijn, the Netherlands) containing 25 mL Production Medium (PM; SCMM in which L‐asparagine monohydrate was replaced by 1.31 g (NH_4_)_2_SO_4_ (Sigma‐Aldrich, St Louis, MO, USA). After 4 days, 25 mL PM was added (total volume of 50 mL) and the mycelium was macerated for 30 s at 18000 rpm in a Waring 2 Speed Blender (Eberbach, USA) using a stainless steel Semi‐Micro Blending Container (Eberbach, USA). The macerate was grown at 30 °C and 200 rpm in a 250 mL Erlenmeyer flask (Thermo Fisher Scientific, Waltham, Massachusetts, USA) in an Innova S44i incubator (Eppendorf, Hamburg, Germany). The mycelium was again macerated after 24 h of culturing and a total of 2 g L^−1^ wet weight mycelium was transferred to a 100 mL Erlenmeyer flask (Thermo Fisher Scientific, Waltham, Massachusetts, USA) containing 20 mL labeled l‐PM (PM, in which the carbon and nitrogen source were replaced for 20 g ^13^C_6_‐glucose (Cambridge Isotope Laboratories, Tewksbury, MA, USA), and 1.31 g (^15^NH_4_)_2_SO_4_ (Cambridge Isotope Laboratories, Tewksbury, MA, USA). Cultures were grown for 6 days at 200 rpm at 30 °C.

**Solid‐State NMR sample preparation**: Mycelium was harvested from l‐PM by centrifugation (13751 rcf, for 5 minutes), freeze dried, frozen in liquid nitrogen and homogenized with mortar and pestle. The homogenate was washed 4 times with 40 mL ultra‐pure water, each time followed by centrifugation at 4951 rcf for 10 minutes. Three aliquots of 10 mg (wet weight) cell walls were incubated at room temperature in 1 mL ultra‐pure water containing 0.74 mM or 18.5 mM Cu(II), or 5 μM Cathelicidin‐2 (Fmoc, HPLC, 95 % purity, ChinaPeptides, Shanghai, China). Cell walls were pelleted at 4951 rcf for 10 minutes and washed three times with ultra‐pure water, each step followed by centrifugation at 4951 rcf for 10 minutes. Samples were resuspended in 1 mL of ultra‐pure water and subjected to ssNMR spectroscopy experiments. Cell walls that had not been incubated with ligands served as a control. *S. commune* strain H4‐8A similarity to strain H4‐39 used in Ehren et al.^[10d]^ was confirmed via 2D ^13^C‐^13^C experiments (data not shown). The 1.3 mm rotor caps and lids used for ^1^H‐detected experiments were sealed with nail polish to avoid sample dehydration during fast MAS.

**Solid‐state NMR spectroscopy experiments**: The scalar‐based ^1^H‐detected NMR experiments of the (^13^C,^15^N)‐labeled *S. commune* with CC‐mixing were carried out at 260 K with 60 kHz magic angle spinning (MAS) on a narrow bore 1.2 GHz (28.2 T) spectrometer using a 1.3 mm HXY MAS probe (Bruker BioSpin). WALTZ16^[28]^ decoupling was applied and WALTZ16 C−C mixing was applied for 11 ms. Actual sample temperature due to frictional heating was 293 K, calibrated using a KBr powder sample.^[29]^ The parameters and conditions for all acquired experiments are summarized in Table S2. Respectively, 120 ms and 200 ms of MISSISSIPPI^[30]^ of water suppression was applied during dipolar‐based and scalar‐based experiments. ^1^H‐detected NMR experiments of (^13^C,^15^N)‐labeled *S. commune* cell wall apo (blank), Cu(II)‐bound (0.74 mM and 18.5 mM) and CATH‐2 bound samples were acquired on a narrow‐bore 700 MHz (16.5 T) spectrometer using a 1.3 mm HXY MAS probe (Bruker BioSpin). Actual sample temperatures due to frictional heating were roughly 298 K, calibrated using a KBr powder sample.^[29]^ WALTZ16^[28]^ decoupling on ^1^H and ^13^C channels was applied during scalar‐based experiments.^[31]^ The ^13^C offset for all scalar‐based experiments was set at 51 ppm with a spectral width of 130 ppm. For dipolar‐based hCH experiments^[32]^ that result in ^1^H‐^13^C 2D correlation spectra, ramped (70 %) forward cross‐polarization from ^1^H to ^13^C with a contact time of 1.2 ms were applied as well as ramped (70 %) backwards cross‐polarization with a short 0.2 ms contact time to ensure that only correlations from directly bonded proton‐carbon pairs would arise. PISSARRO^[33]^ decoupling on ^1^H and ^13^C channels was applied during dipolar‐based experiments. The ^13^C offset for all dipolar‐based experiments was set at 57.3 ppm with a spectral width of 130 ppm. Carbon‐detected CP‐based Double Quantum Single Quantum^[21]^ (DQSQ) ^13^C−^13^C 2D correlation spectra were recorded on a narrow bore 700 MHz spectrometer using a 3.2 mm HCN Efree MAS probe (Bruker BioSpin). An SPC5^[21]^ mixing time of 0.4 ms was applied with CW decoupling on the ^1^H channel during excitation and reconversion and SPINAL64 decoupling was applied on the ^1^H channel during acquisition. The ^13^C offset was set to 86 ppm with a spectral width of the 304 ppm in the direct dimension and 280 ppm in the indirect dimension.

A detailed description of the solid‐state NMR data acquisition parameters and analysis is provided in the Supporting Information.

## Conflict of interest

The authors declare no conflict of interest.

1

## Supporting information

As a service to our authors and readers, this journal provides supporting information supplied by the authors. Such materials are peer reviewed and may be re‐organized for online delivery, but are not copy‐edited or typeset. Technical support issues arising from supporting information (other than missing files) should be addressed to the authors.

Supporting Information

## Data Availability

The data that support the findings of this study are available from the corresponding author upon reasonable request.
