# Supplementary material for: Probing Cell‐Surface Interactions in Fungal Cell Walls by High‐Resolution 1H‐Detected Solid‐State NMR Spectroscopy
Source: Chemistry. 2022 Nov 10;29(1):e202202616. doi: 10.1002/chem.202202616 (PMC10099940; doi:10.1002/chem.202202616)
Supplement: Supplementary file 1 — Supporting Information [file CHEM-29-0-s001.pdf]

Chemistry–A European Journal

Supporting Information

Probing Cell-Surface Interactions in Fungal Cell Walls by High-Resolution ^{1}H-Detected Solid-State NMR Spectroscopy

Adil Safeer, Fleur Kleijburg, Salima Bahri, David Beriashvili, Edwin J. A. Veldhuizen, Jacq van Neer, Martin Tegelaar, Hans de Cock, Han A. B. Wösten,* and Marc Baldus*

Supplementary Information

Probing Cell-Surface Interactions in Fungal Cell Walls by High-Resolution ^{1}H-detected Solid-State NMR Spectroscopy

This PDF file includes:

- Figures S1 to S11
- Tables S1 to S12
- Supplementary text
- SI References
- Author contributions

Supplementary information figures

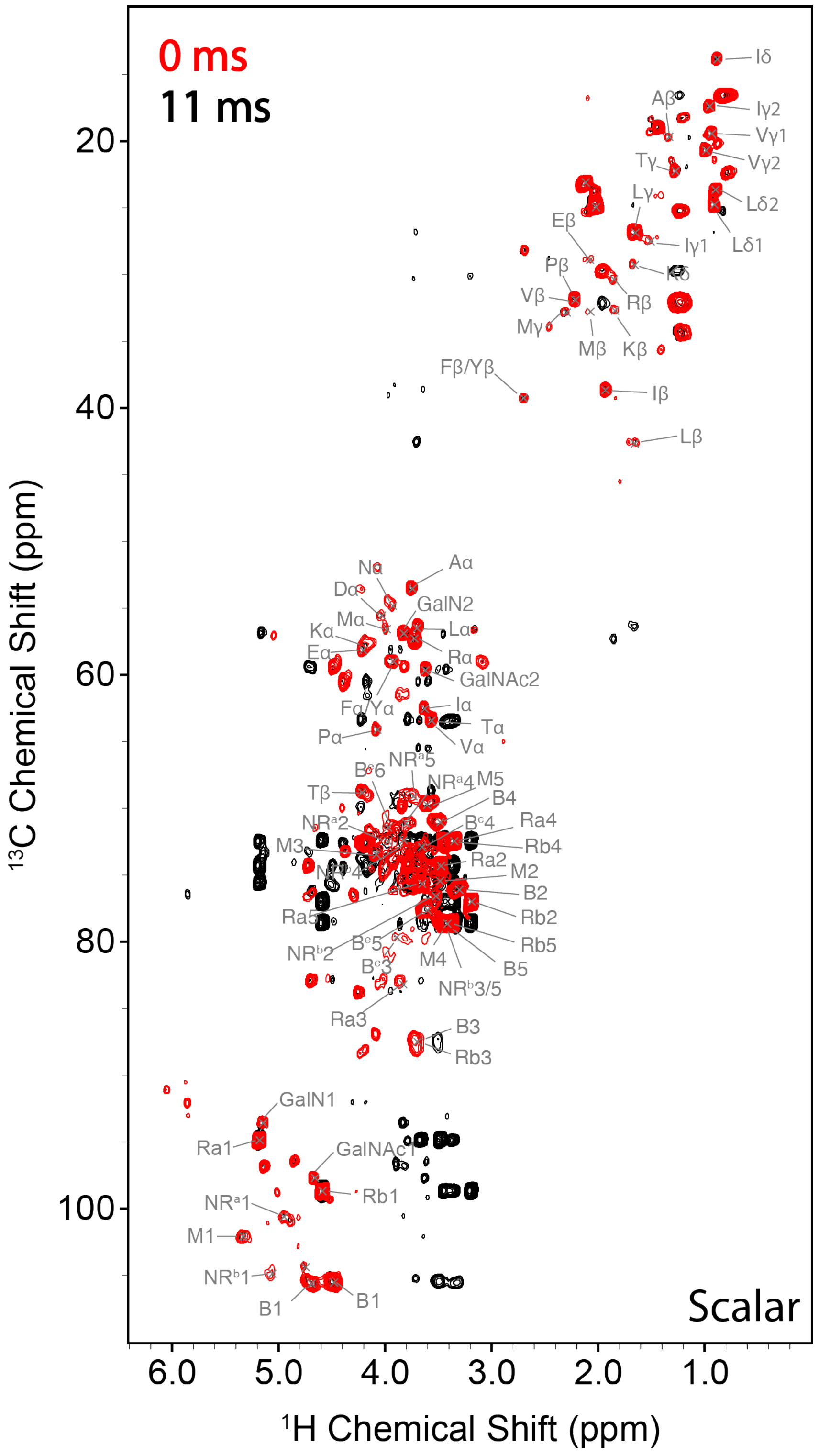

Figure S1. Overlay of scalar-based ^{1}H-^{13}C correlation spectra with 0 ms (red) and 11 ms (black) of WALTZ16 CC mixing.

A

```
1           10          20          30         40          50           60          70          80
MFARLPVVFLYAFVAFGALVAALPGGHPGTTTPPVTTTVTVTTPPSTTTIAAGGTCTTGSLSCCNQVQSASSSPVTALLGLL
        90          100         110         120         130
GIVLSDLNVLVGISCSPLTVIGVGGSGCSAQTVCCENTQFNGLINIGCTPINIL
```

B

```
          10          20
RFGRFLRKIRRFRPKVTITIQGSARF-NH2
```

Figure S2. Amino acid sequences of (**A**) the hydrophobin SC3[1] and (**B**) antifungal peptide CATH-2[2].

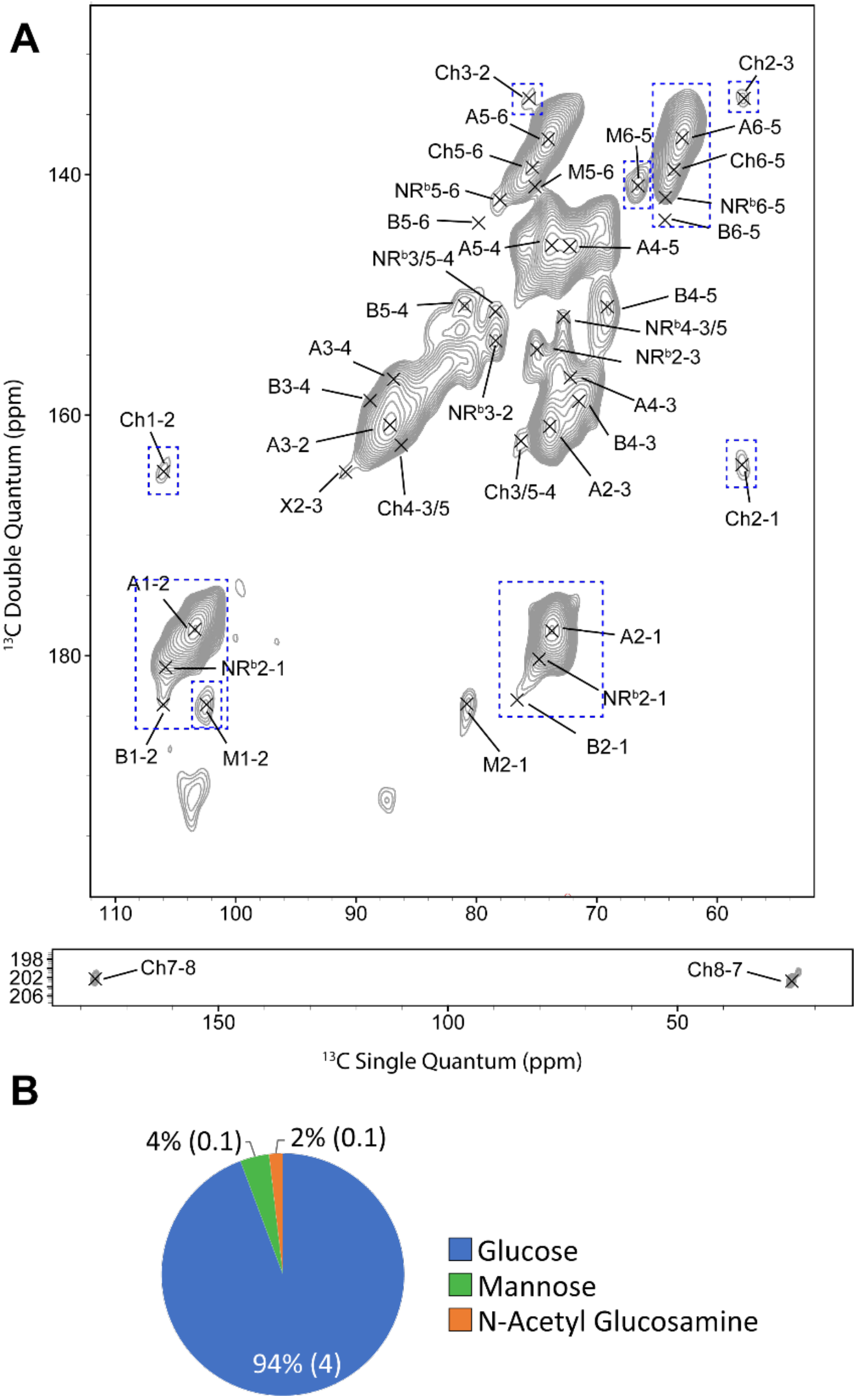

Figure S3. Relative contribution of polysaccharides to the rigid *S. commune* cell wall as probed by ^{13}C-detected ssNMR. (**A**) Two-dimensional Double Quantum-Single Quantum ^{13}C-^{13}C correlation spectrum of the *S. commune* cell wall. The dashed boxes (blue) represent integration areas used to determine peak volume (Table S8, Vide Infra). (**B**) Pie chart displaying a breakdown of the relative monomer contribution to the rigid cell wall. Calculated error is shown in brackets next to the percentages.

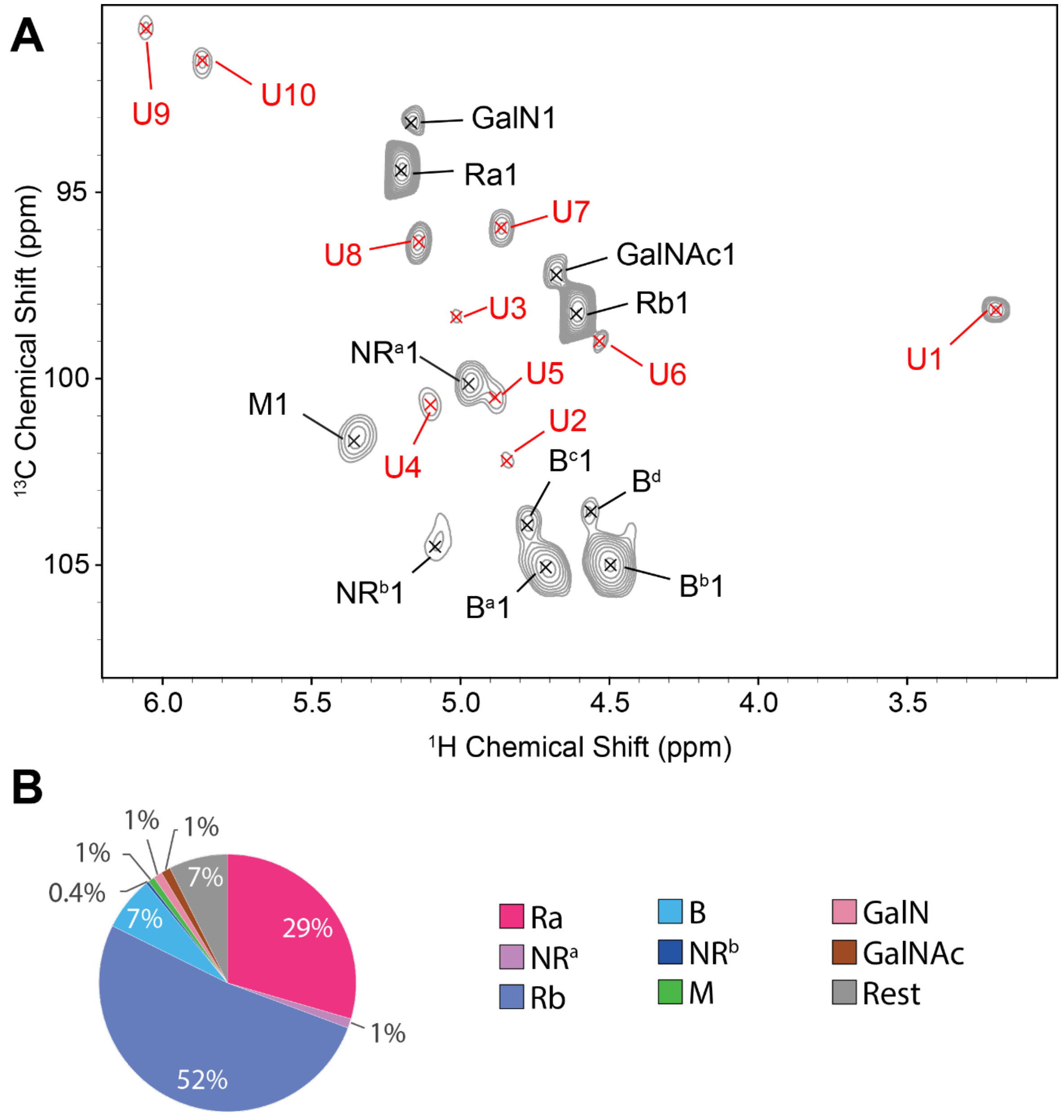

Figure S4. Relative contribution of polysaccharides to the mobile *S. commune* cell wall as probed by a ^{1}H-detected ssNMR. (**A**) The defined carbohydrate C1 region from a scalar based ^{1}H-^{13}C 2D correlation spectrum of the *S. commune* cell wall. Unassigned resonances are labeled 'U'. (**B**) Pie chart displaying a breakdown of the relative contribution of the polysaccharide species to the flexible cell wall (Table S11, Vide Infra). β-(1,3)-glucan ($B^{a,b,c,d}$: light blue); non-reducing end β-(1,3)-glucan (NR^{b}: dark blue); reducing end β-(1,3)-glucan (Rb: indigo); mannan (M: green); non-reducing end α-glucan (NR^{a}: lavender); reducing end α-glucan (Ra: Pink); N-acetyl galactosamine-containing polysaccharides (GalNAc: brown); galactosamine-containing polysaccharides (GalN; pink).

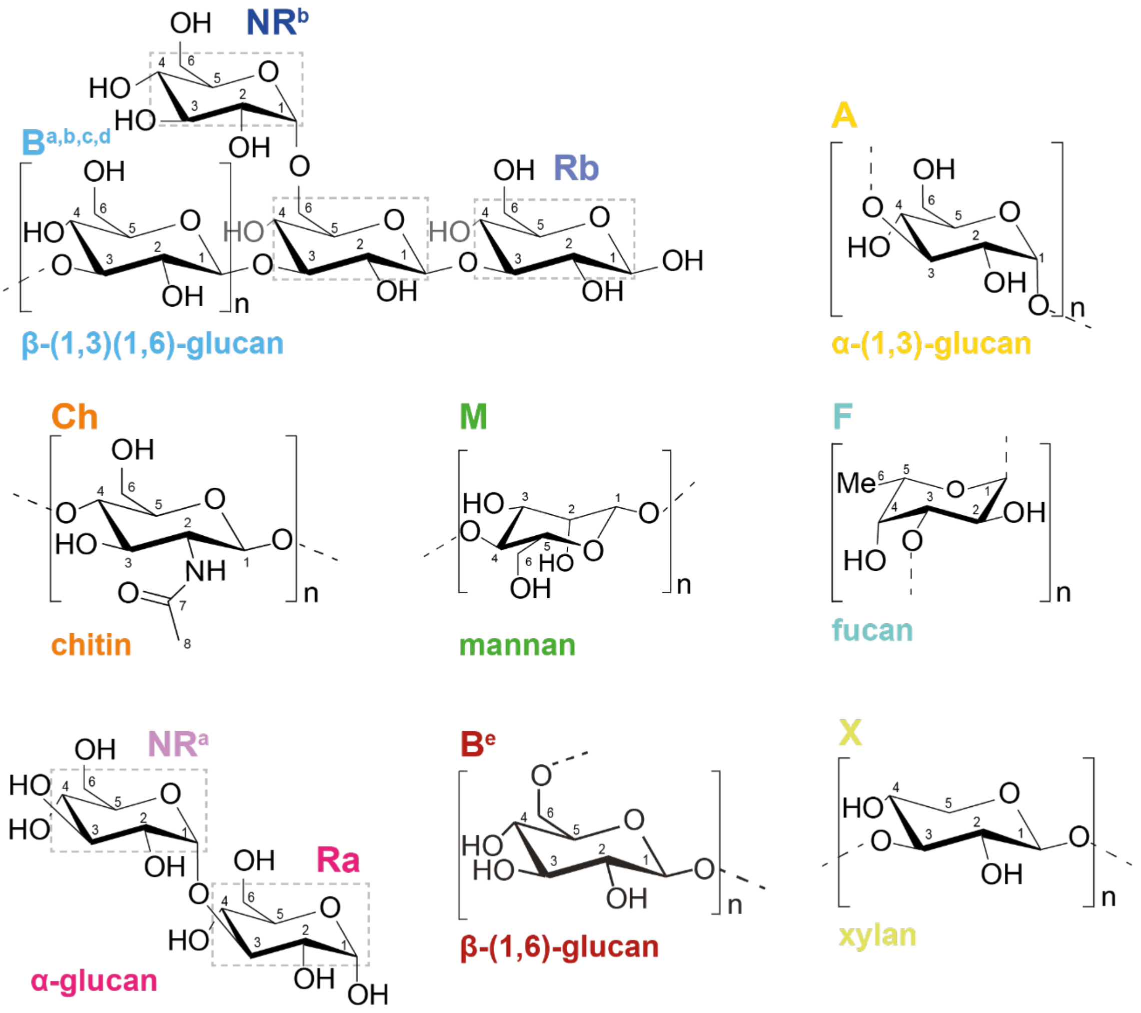

Figure S5. Molecular structures of previously identified[3] *S. commune* cell wall polysaccharides: β-(1,3)-glucan (B[a,b,c,d]: light blue); non-reducing end β-(1,3)-glucan (NR[b]: dark blue); reducing end β-(1,3)-glucan (Rb: indigo); α-(1,3)-glucan (A: gold); chitin (Ch: orange); mannan (M: green); fucan (F: teal); non-reducing end α-glucan (NR[a]: lavender); reducing end α-glucan (Ra: Pink); β-(1,6)-glucan (B[e]: dark red); xylan (X: lime). Numbers correspond to saccharide carbons.

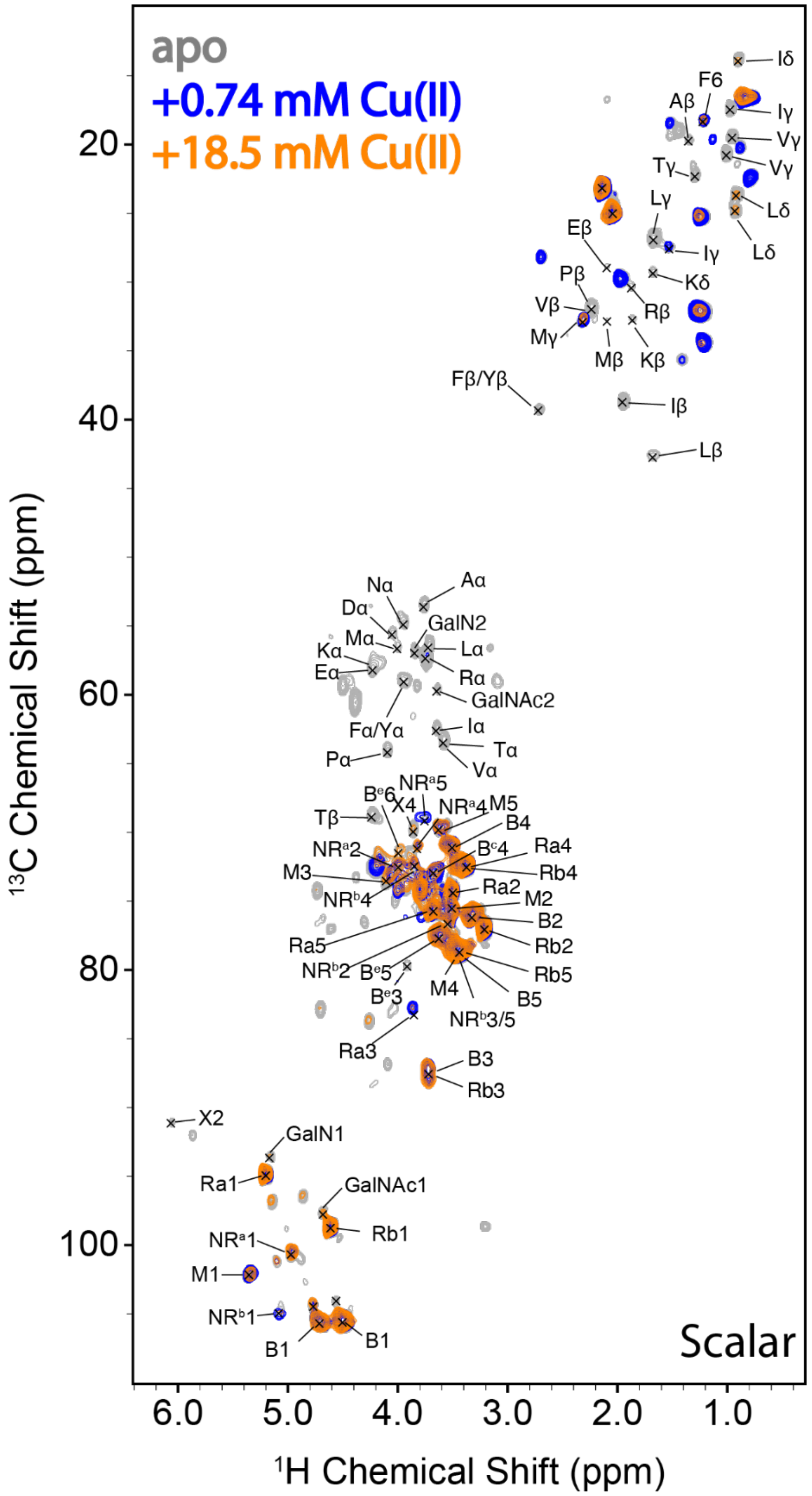

Figure S6. Overlay of scalar-based ^{1}H-^{13}C 2D correlation spectra displaying changes upon the addition of 0.74 mM Cu(II) (blue) and 18.5 mM Cu(II) (orange).

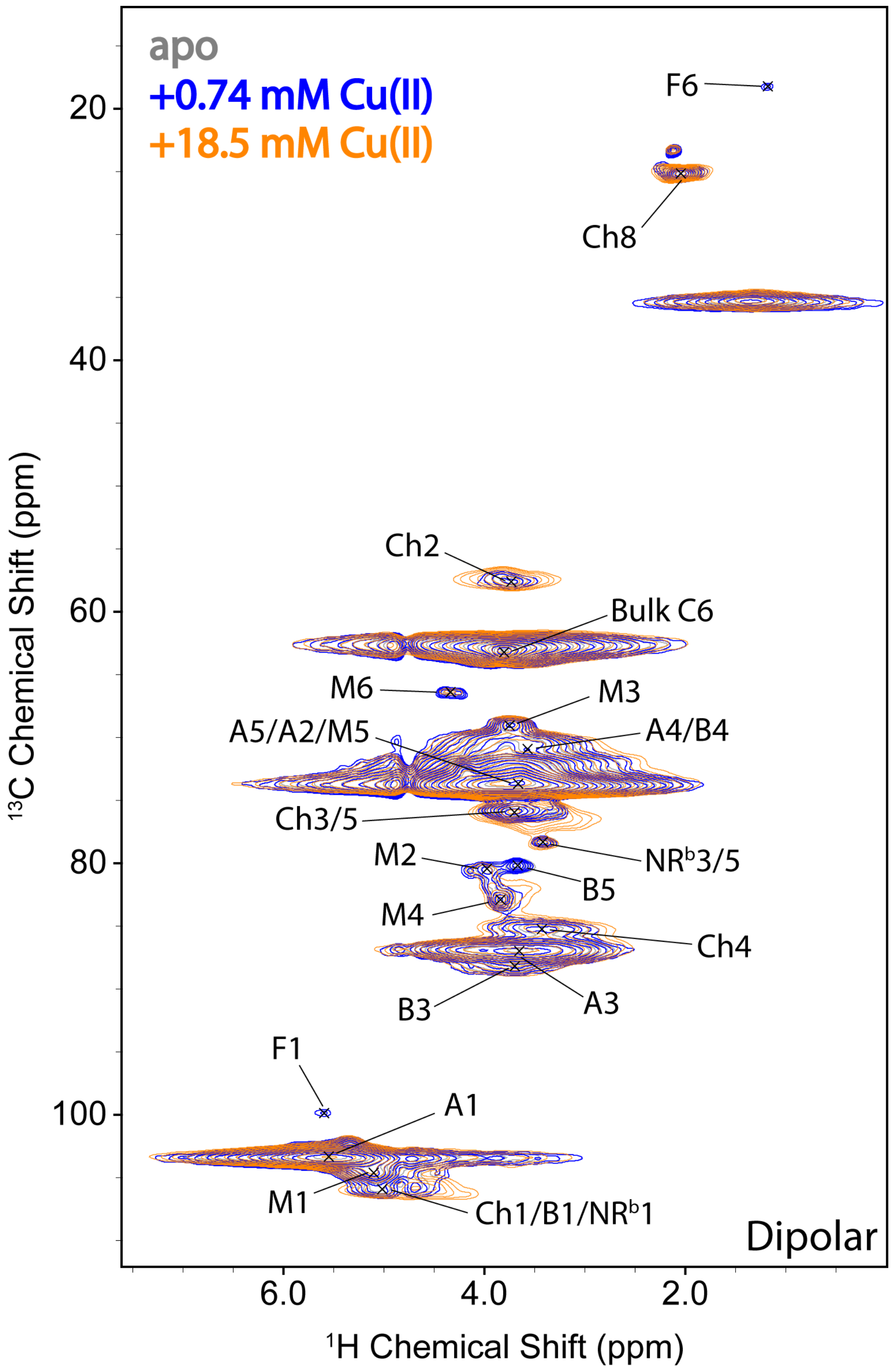

Figure S7. Overlay of dipolar-based ^{1}H-^{13}C 2D correlation spectra displaying changes upon the addition of 0.74 mM Cu(II) (blue) and 18.5 mM Cu(II) (orange).

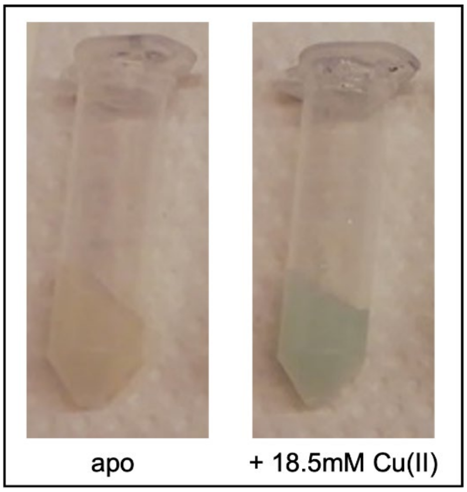

Figure S8. Color staining of the *S. commune* cell wall upon binding by Cu(II) ions: blank cell walls (left), cell walls after addition of 18.5mM Cu(II) (right).

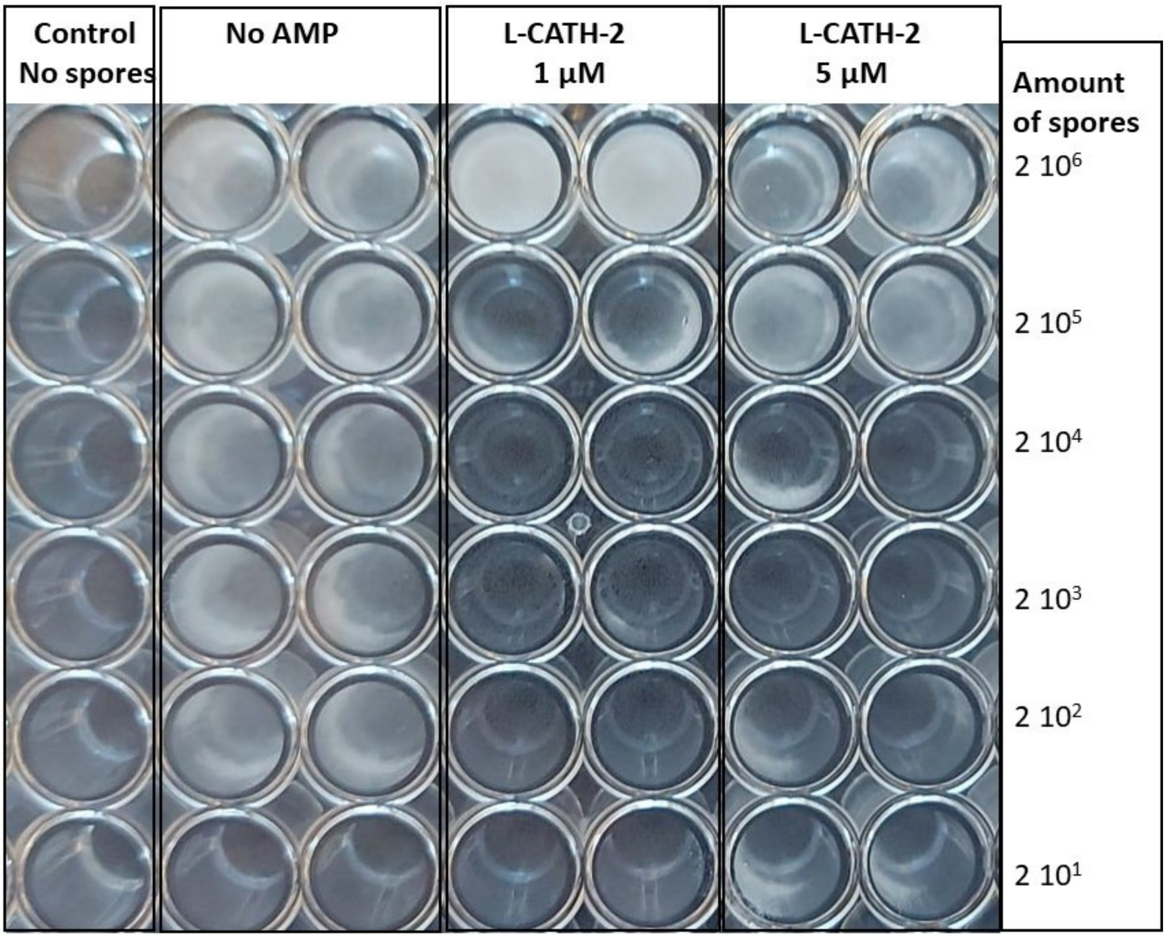

Figure S9. *S*. *commune* spore killing after incubation with 5 µM Cathelicidin-2.

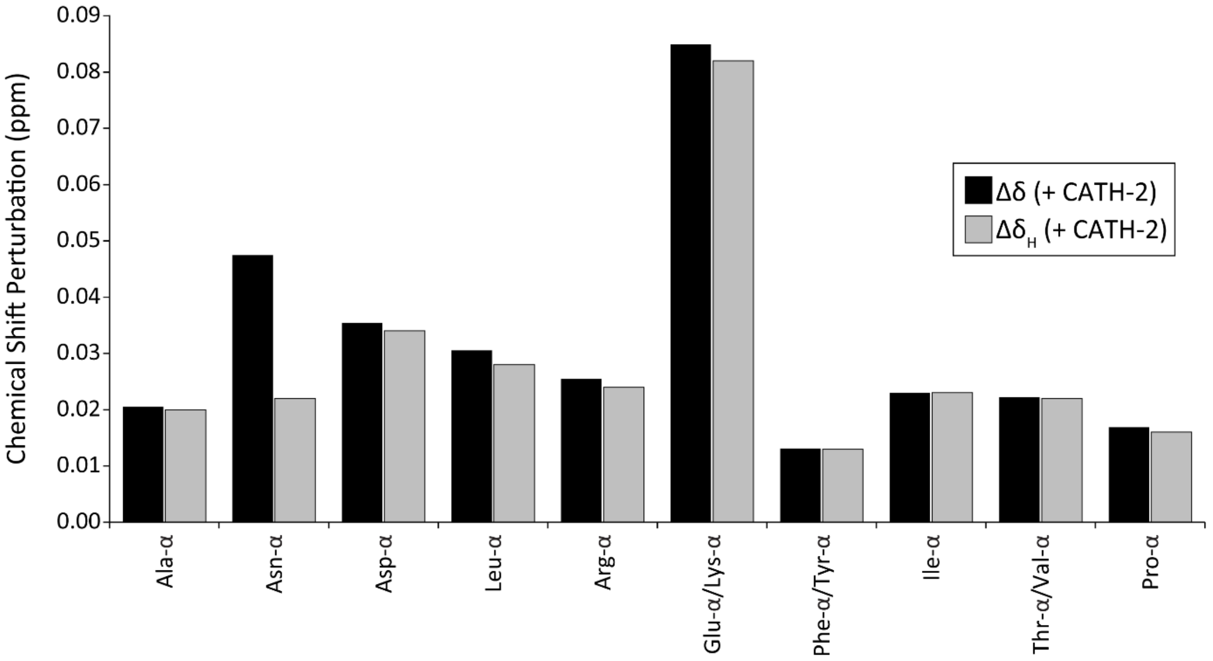

Figure S10. Weighted Chemical Shift Perturbations (CSPs) calculated from peak-shifts that occur in the scalar-based ^{1}H-^{13}C 2D correlation spectra upon the addition of Cathelicidin-2. Grey bars display CSPs only taking into account perturbations in the ^{1}H dimension.

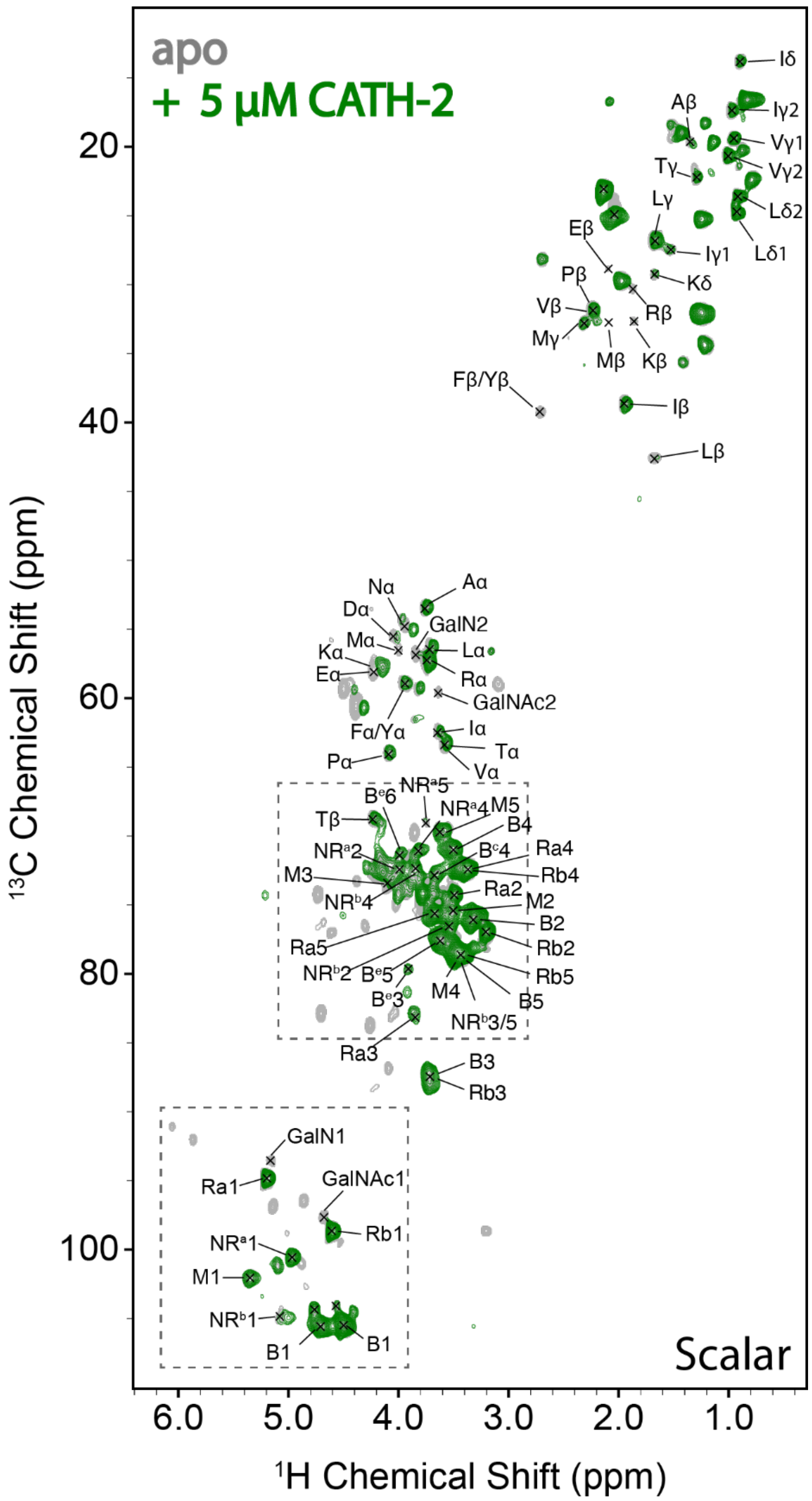

Figure S11. Overlay of scalar-based ^{1}H-^{13}C 2D correlation spectra displaying changes upon the addition of 5 µM antifungal peptide CATH-2 to the *S. commune* cell wall. The boxes denote the bulk polysaccharide region in which changes also occur.

Supplementary information tables

Table S1. Improvement of proton linewidth at 1.2 GHz versus 700 MHz.

Resonance	700 MHz ^{1}H peak width (ppm)	700 MHz ^{1}H peak width (Hz)	1.2 GHz ^{1}H peak width (ppm)	1.2 GHz ^{1}H peak width (Hz)	Improvement (based on ppm)
Ch8	0.32	226	0.17	203	1.9
Bulk C6	0.49	345	0.24	285	2.1
M4	0.34	238	0.15	183	2.2
A1	0.97	678	0.58	692	1.7

Numbers represent ^{1}H full peak width at half height (fwhh).

List of abbreviations:

Ch8: C8 of chitin
Bulk C6: C6 of all rigid polysaccharides in system
M4: C4 of branched mannan
A1: C1 of α-(1,3)-glucan

Table S2. Acquisition parameters and conditions for solid-state NMR experiments that were recorded of the *S. commune* cell wall.

Sample	Exp	NS	DS	D1 (s)	^{1}H 90° (kHz)	^{13}C 90° (kHz)	^{1}H dec. (kHz)	^{13}C dec (kHz)	H_2O supp (kHz)	Max F1 ^{1}H acqu time (ms)	Max F2 ^{13}C acqu time (ms)	B_0 (MHz)	MAS (kHz)	Set T (K)
apo	Scalar 2D CH	96	32	1.4	100	67	10	10	17	29.3	5.09	1200	60	260
	Dipolar 2D CH	64	16	1.4	100	67	10	11	21	20.0	3.82	1200	60	260
apo	Scalar 2D CH	96	32	1.4	156	92	10	10	23	29.3	8.74	700	60	258
	Dipolar 2D CH	64	16	1.4	156	92	10	10	23	19.9	6.55	700	60	256
	Dipolar 2D CH	352	16	1.0	156	92	10	10	23	19.9	6.55	700	60	258
+ 0.74 mM Cu(II)	Scalar 2D CH	96	32	32	156	93	10	10	23	29.3	8.74	700	60	256
	Dipolar 2D CH	352	16	1.0	156	93	10	10	23	19.9	6.55	700	60	256
+ 18.5 mM Cu(II)	Scalar 2D CH	96	32	1.4	156	93	10	10	23	29.3	8.74	700	56	259
	Dipolar 2D CH	352	16	1.0	156	93	10	10	23	19.9	6.55	700	56	259
+ 5μM CATH-2	Scalar 2D CH	96	32	1.4	156	93	10	10	23	29.3	8.74	700	60	259

Sample	Exp	NS	DS	D1 (s)	^{1}H 90° (kHz)	^{13}C 90° (kHz)	^{1}H dec. (kHz)	SPC 5 ^{13}C (kHz)	^{1}H CW dec. (kHz)	Max F1 ^{13}C Acqu time (ms)	Max F2 ^{13}C Acqu time (ms)	B_0 (MHz)	MAS (kHz)	Set T (K)
apo	CP-DQSQ 2D CC	416	32	2.0	81	49	81	40	95	9.55	2.53	700	10	260

List of abbreviations:

apo: blank sample
Exp: type of experiment
NS: number of scans
DS: number of dummy scans
D1: recycle delay
$^{1}H/^{13}C$ 90°: applied $^{1}H/^{13}C$ hard 90 pulses applied
$^{1}H/^{13}C$ dec: applied $^{1}H/^{13}C$-channel decoupling power
H_2O supp: applied water-suppression power
Max acqu time: maximum acquisition time
B_0: magnetic field strength
MAS: magic angle spinning rate
Set T: set temperature

Table S3. Assignments of polysaccharide signals in the dipolar-based ^1H-^{13}C correlation spectra. ^{13}C chemical shifts were previously[3] assigned for this system. ^1H assignments were obtained by following one-bond correlations and by cross-validation using database[9] and earlier literature values. Relevant literature values are cited in brackets.

Species	C1 (ppm)<br>H1 (ppm)	C2 (ppm)<br>H2 (ppm)	C3 (ppm)<br>H3 (ppm)	C4 (ppm)<br>H4 (ppm)	C5 (ppm)<br>H5 (ppm)	C6 (ppm)<br>H6 (ppm)	C8 (ppm)<br>H8 (ppm)
A	103.4<br>5.54[4]	73.6<br>3.66	87.0<br>3.61	73.6<br>3.66	73.6<br>3.66	63.1<br>3.80	
B	105.9<br>5.07	75.6<br>3.39	88.7<br>-	70.5<br>3.51	80.2<br>3.66[5]	63.1<br>3.80	
NR[b]		75.6<br>3.39	78.3<br>3.42[5]		78.3<br>3.42[5]	63.1<br>3.80	
Ch	105.9<br>5.07	57.6<br>3.73	75.8<br>3.72	85.2<br>3.43[6]	75.8<br>3.72	63.1<br>3.80	25.2<br>2.05
M	104.9<br>-	80.5<br>3.97[7]		69.0<br>3.74[7]		66.4<br>4.26[7]	
F	99.8<br>5.62[8]					18.2<br>1.18	

List of abbreviations:

A: α-(1,3)-glucan
B: β-(1,3)-glucan
NRb: non reducing end β -(1,3)-glucan
Ch: chitin
M: branched mannose
F: fucan

Table S4. Assignments of polysaccharide signals in the scalar-based ^{1}H-^{13}C correlation spectra. ^{13}C chemical shifts were previously[3] assigned for this system. ^{1}H assignments were obtained by following one-bond correlations and by cross-validation using database[9] and earlier literature values. Relevant literature values are cited in brackets. Ambiguous ^{1}H chemical shifts are italicized.

**Species**	**C1 (ppm)<br>H1 (ppm)**	**C2 (ppm)<br>H2 (ppm)**	**C3 (ppm)<br>H3 (ppm)**	**C4 (ppm)<br>H4 (ppm)**	**C5 (ppm)<br>H5 (ppm)**	**C6 (ppm)<br>H6 (ppm)**
B^{a}	105.7<br>4.71[5]	76.0<br>3.31	87.2<br>3.73	71.0<br>3.50	78.6<br>3.44	
B^{b}	105.5<br>4.50					
B^{c}	104.4<br>4.77					
B^{d}	104.1<br>4.57					
Rb	98.7<br>4.61[5]	77.0<br>3.20	87.2<br>3.73	72.4<br>3.37	78.6<br>3.44	
NR^{b}	104.9<br>5.08	76.5<br>3.54	78.6<br>3.44	72.3<br>*3.85*	78.6<br>3.44[5]	
B^{e}			79.7<br>3.91	72.8<br>3.68	77.5<br>3.61	71.3<br>*3.99*
Ra	94.9<br>5.19[5]	74.3<br>3.49	82.8<br>3.87	72.4<br>3.37	75.6<br>3.67	
NR^{a}	100.6<br>4.96	72.4<br>*3.99*		71.0<br>*3.82*	69.0<br>3.75[10]	
M	102.1<br>5.35	75.4<br>3.50	73.3<br>4.10	78.6<br>3.44	69.6<br>3.62[7]	
GalNAc	97.7<br>4.67[11]	59.5<br>3.64				
GalN	93.6<br>5.16	56.8<br>3.84				

List of abbreviations:

$B^{a,b,c,d}$: β-(1,3)-glucan
Rb: reducing end β-(1,3)-glucan
NR^{b}: non reducing end β-(1,3)-glucan
B^{e}: β-(1,6)-glucan
Ra: reducing end α-(1,3)-glucan
NR^{a}: non reducing end α-(1,3)-glucan
M: mannan
GalNAc: N-acetyl galactosamine
GalN: galactosamine

Table S5. Assignments of amino-acid signals in the scalar-based ^{1}H-^{13}C correlation spectra. ^{13}C chemical shifts were previously[3] assigned for this system. ^{1}H assignments were obtained by following one-bond correlations and by cross-validation using database[12] and earlier literature values[13].

**Amino acid**	**Cα (ppm)**<br>**Hα (ppm)**	**Cβ (ppm)**<br>**Hβ (ppm)**	**Cγ (ppm)**<br>**Hγ (ppm)**	**Cδ (ppm)**<br>**Hδ (ppm)**
Ala (A)	53.5<br>3.76	19.6<br>1.35		
Arg (R)	57.2<br>3.74	30.3<br>1.87		
Asn (N)	54.7<br>3.94			
Asp (D)	55.5<br>4.04	39.3<br>1.84		
Glu (E)	58.1<br>4.23	28.8<br>2.10		
Ile (I)	62.4<br>3.64	38.6<br>1.94	27.4; 17.4<br>1.53; 0.97	13.8<br>0.90
Leu (L)	56.3<br>3.71	42.6<br>1.68	26.8<br>1.67	23.6; 24.8<br>0.91; 0.92
Lys (K)	58.1<br>4.23	32.6<br>1.86	24.0<br>1.42	29.2<br>1.67
Met (M)	56.5<br>4.00	32.7<br>2.09	32.8<br>2.32	
Phe/Tyr<br>(F/Y)	58.9<br>3.95	39.2<br>2.71		
Pro (P)	64.0<br>4.09	31.8<br>2.23		
Thr (T)	63.3<br>3.58	68.7<br>4.23	22.2<br>1.29	
Val (V)	63.3<br>3.58	31.8<br>2.23	19.4; 20.7<br>0.95; 1.00	

Table S6. Volumes of defined integration areas and the relative contributions to the full *S. commune* rigid cell wall polysaccharide volume.

Integral #	Contribution per integrated peak	Integral volume	Relative contribution
1	A2/A4/A5/B2/B4/M4/M5/NR^{b}2/NR^{b}4/F2/F3/F4/F5/Ch3/Ch5	$9.09 \cdot 10^9$	52%
2	C6 (A/B/Ch/NR^b)	$2.33 \cdot 10^9$	13%
3	C1 (A/B/Ch/M/NRb)	$2.91 \cdot 10^9$	17%
4	Ch4/A3/B3	$1.94 \cdot 10^8$	11%
5	Ch8	$1.91 \cdot 10^8$	1.1%
6	U	$2.23 \cdot 10^8$	1.3%
7	B5	$1.27 \cdot 10^8$	0.73%
8	Ch2	$1.57 \cdot 10^8$	0.91%
9	M6	$7.50 \cdot 10^7$	0.43%
10	NR^{b}3/5	$1.31 \cdot 10^8$	0.75%
11	M2	$1.29 \cdot 10^8$	0.74%
12	F6	$2.02 \cdot 10^7$	0.12%
13	F1	$5.22 \cdot 10^7$	0.30%
	**Total**	$1.74 \cdot 10^{10}$	100%

List of abbreviations:

A: α-(1,3)-glucan
B: β-(1,3)-glucan
NR^b: non reducing end β-(1,3)-glucan
Ch: chitin
M: branched mannan
F: fucan
U: unknown

Numbers represent saccharide carbons: C1, C2, C3, C3, C4, C5, C6, C7 and C8

Table S7. Relative contributions of defined polysaccharide species to the rigid *S. commune* cell wall.

Resolved peaks	Polysaccharide species	Average rel. contribution	As % of rigid cell wall polysaccharides
Ch(2,8)	Ch	1.0%	1.0% * 7 CH pairs = 7.0%
M(2,6)	M	0.82%	0.82% * 6 CH pairs = 4.9%
F(1,6)	F	0.21%	0.21% * 6 CH pairs = 1.2%
B(5)	B	0.73%	0.73% * 6 CH pairs = 4.4%
NR^b(3,5)	NR^b	0.38%	0.38% * 6 CH pairs = 2.3%
> Solve α-(1,3)-glucan	A	9.5%	9.5% * 6 CH pairs = 57%
	**Total**		77%
	Rest		23%

List of abbreviations:

A: α-(1,3)-glucan
B: β-(1,3)-glucan
NR^b: non reducing end β-(1,3)-glucan
Ch: chitin
M: branched mannan
F: fucan

Numbers represent saccharide carbons C1, C2, C3, C3, C4, C5, C6, C7 and C8

Table S8. Volumes of defined integration areas in the Double Quantum Single Quantum ^{13}C-^{13}C 2D spectrum of the rigid *S. commune* cell wall.

Integral #	Contribution per integrated peak	Integral volume (abs)
1	Ch7-8	$3.76 \cdot 10^{6}$
2	Ch8-7	$6.96 \cdot 10^{6}$
3	M2-1	$3.80 \cdot 10^{6}$
4	A2-1/NR^b2-1/B2-1	$1.26 \cdot 10^{8}$
5	A1-2/NR^b1-2/B1-2	$1.44 \cdot 10^{8}$
6	Ch2-1	$3.11 \cdot 10^{6}$
7	Ch1-2	$2.30 \cdot 10^{6}$
8	Ch2-3	$1.66 \cdot 10^{6}$
9	Ch6-5/A6-5/NR^b6-5/B6-5	$9.44 \cdot 10^{7}$
10	M6-5	$5.37 \cdot 10^{6}$
11	M1-2	$4.93 \cdot 10^{6}$
12	Ch3-2	$2.20 \cdot 10^{6}$

List of abbreviations:

A: α-(1,3)-glucan
B: β-(1,3)-glucan
NR^b: non reducing end β-(1,3)-glucan
Ch: chitin
M: branched mannan

Numbers represent saccharide carbons: C1, C2, C3, C3, C4, C5, C6, C7 and C8 (see Fig. S3)

Table S9. Relative monomer contribution to the rigid *S. commune* cell wall.

Spin	Abs area	Relative monomer abundance	Error
N-Acetyl Glucosamine C2	$3.11 \cdot 10^6$		
N-Acetyl Glucosamine C1	$2.30 \cdot 10^6$		
N-Acetyl Glucosamine C1	$1.66 \cdot 10^6$		
N-Acetyl Glucosamine C3	$2.20 \cdot 10^6$	1.8%	0.097%
Mannose C6	$5.37 \cdot 10^6$		
Mannose C2	$3.80 \cdot 10^6$		
Mannose C1	$4.93 \cdot 10^6$	3.7%	0.13%
Glucose C1	$1.40 \cdot 10^8$		
Glucose C2	$1.26 \cdot 10^8$		
Glucose C6	$9.21 \cdot 10^7$	94%	4%

Table S10. Intensities of peaks in the carbohydrate C1 region of the scalar-based ^{1}H-^{13}C 2D correlation spectra of *S. commune* cell wall. Peaks labeled U represent unassigned resonances.

Peak	^{1}H (ppm)	^{13}C (ppm)	Intensity (abs)	Rel Int.
U1	3.20	98.6	$3.52 \cdot 10^6$	1.4%
U2	4.84	102.7	$6.93 \cdot 10^5$	0.27%
U3	5.01	98.8	$6.98 \cdot 10^5$	0.27%
NR^{b}1	5.07	104.9	$8.96 \cdot 10^5$	0.35%
U4	5.10	101.2	$1.14 \cdot 10^6$	0.45%
U5	4.89	101.0	$1.20 \cdot 10^6$	0.47%
B^{d}1	4.57	104.0	$1.25 \cdot 10^6$	0.49%
U6	4.53	99.5	$1.59 \cdot 10^6$	0.62%
B^{c}1	4.77	104.4	$2.00 \cdot 10^6$	0.78%
M1	5.35	102.1	$2.16 \cdot 10^6$	0.85%
U7	4.86	96.5	$2.60 \cdot 10^6$	1.0%
GalN1	5.16	93.6	$2.84 \cdot 10^6$	1.1%
GalNAc1	4.67	97.7	$3.04 \cdot 10^6$	1.2%
NR^{a}1	4.96	100.6	$3.06 \cdot 10^6$	1.2%
U8	5.14	96.8	$3.99 \cdot 10^6$	1.6%
B^{b}1	4.70	105.6	$5.45 \cdot 10^6$	2.1%
B^{a}1	4.50	105.5	$9.85 \cdot 10^6$	3.9%
Ra1	5.20	94.9	$7.53 \cdot 10^7$	29%
Rb1	4.60	98.7	$1.32 \cdot 10^8$	52%
U9	6.06	91.1	$9.23 \cdot 10^5$	0.36%
U10	5.87	92.0	$1.34 \cdot 10^6$	0.52%
**Total**			$2.55 \cdot 10^8$	100%

List of abbreviations:

B^{a,b,c,d}: β-(1,3)-glucan
Rb: reducing end β-(1,3)-glucan
NR^b: non reducing end β-(1,3)-glucan
Ra: reducing end α-(1,3)-glucan
NR^a: non reducing end α-(1,3)-glucan
M: mannan
GalNAc: N-acetyl galactosamine
GalN: galactosamine

Numbers represent saccharide C1 carbons

Table S11. Relative contributions of defined polysaccharide species to the flexible *S. commune* cell wall.

Polysaccharide species	As % of flexible cell wall polysaccharides
α-(1,3)-glucan	31%
Ra	29%
NR^{a}	1.2%
β-(1,3)/(1,6)-glucan	59%
Rb	52%
$B^{a,b,c,d}$	7.3%
NR^{b}	0.35%
Mannan	0.85%
GalN	1.1%
GalNAc	1.2%
Rest (U)	6.9%
Total	100%

List of abbreviations:

Rb: reducing end β-(1,3)-glucan
$B^{a,b,c,d}$: β-(1,3)-glucan
NR^{b}: non reducing end β-(1,3)-glucan
Ra: reducing end α-(1,3)-glucan
NR^{a}: non reducing end α-(1,3)-glucan
M: mannan
GalN: galactosamine
GalNAc: N-acetyl galactosamine

Table S12. ^{1}H linewidths at half height of peaks in the carbohydrate C1 region of the scalar-based ^{1}H-^{13}C 2D correlation spectra of *S. commune* cell wall. Peaks labeled 'U' represent unassigned resonances.

**Peak**	**^{1}H peak width (ppm)**	**^{1}H peak width (Hz)**
U1	0.060	41.8
U2	0.077	54.2
U3	0.059	41.4
NR^{b}1	0.135	94.8
U4	0.072	50.7
U5	0.077	54.2
B^{d}1	0.048	33.7
U6	0.035	24.5
B^{c}1	0.062	43.5
M1	0.090	62.9
U7	0.058	40.6
GalN1	0.054	35.4
GalNAc1	0.045	31.7
NR^{a}1	0.073	51.4
U8	0.054	38.0
B^{b}1	0.087	60.6
B^{a}1	0.084	58.7
Ra1	0.058	40.3
Rb1	0.059	41.0
U9	0.056	39.2
U10	0.058	40.3

Numbers represent ^{1}H full peak width at half height (fwhh).

List of abbreviations:

B^{a,b,c,d}: β-(1,3)-glucan
Rb: reducing end β-(1,3)-glucan
NR^{b}: non reducing end β-(1,3)-glucan
Ra: reducing end α-(1,3)-glucan
NR^{a}: non reducing end α-(1,3)-glucan
M: mannan
GalNAc: N-acetyl galactosamine
GalN: galactosamine

Numbers represent saccharide C1 carbons

Supplementary Information Text

Extended Methods

1. Effect of Cathelicidin-2 on the growth of *S. commune*

Spores of *S. commune* strain H4-8A were harvested from mushrooms, suspended in water, counted with a hemocytometer and directly used in growth assays. The indicated number of spores in 150 µL SCMM and 50 mM glucose were incubated for 144 h at 30 °C in presence of 1 or 5 µM CATH-2[2] (Fig. S2). Media without spores or AMP served as control. Fungal growth of *S. commune* after 144 hours in an ELISA plate (Greiner Bio-one F bottom with lid) is shown. Results show inhibition of growth by CATH-2 at spore concentrations of $2 \cdot 10^4$ and lower. At higher spore concentrations partial growth or no growth inhibition was observed.

2. Solid-State NMR experiments

2a. Data analysis

TopSpin version 4.1.0 (Bruker BioSpin) was used to process and analyze the acquired data. Dipolar-based ^{1}H-^{13}C spectra were processed with QSINE SSB 4, and scalar-based ^{1}H-^{13}C spectra were processed with QSINE SSB 2.5, and the TopSpin manual integration function was used for relative abundance analyses. DQSQ ^{13}C-^{13}C 2D spectra were processed with QSINE SSB 3.2. NMRFAM-SPARKY[14] was used to create figures and determine the fwhh of peaks in ^{1}H-detected spectra. Assignments for rigid and flexible cell wall polysaccharides are found in table S3 and S4 respectively. For all shown spectra and assignment tables, the ^{1}H dimension was referenced to the water peak at 4.7 ppm, and the ^{13}C dimension referencing was based on chemical shift values previously published in Ehren *et al*.[3] for the sake of consistency. Importantly, referencing the ^{13}C dimension by water results in a -2.7 ppm difference from the ^{13}C chemical shift values reported here.

2b. Secondary chemical shift calculations

Calculations for the comparison of experimentally observed carbon chemical shifts to standard isotropic α-helix (αh) random coil (rc) and β-strand (βs) values in Figure 1C were performed using a method previously described by Luca *et al*.[15]. The equation is shown below (Vide Infra). Isotropic values were taken from Wang & Jardetzky[13]. and the assignments for flexible cell wall amino acids are shown in table S5.

$$\Delta\delta = \delta C\alpha - \delta C\beta = \{\delta C\alpha\,(\mathrm{obs}) - \delta C\alpha\,(\alpha h/rc/\beta s)\} - \{\delta C\beta\,(\mathrm{obs}) - \delta C\beta\,(\alpha h/rc/\beta s)\}$$

2c. Chemical shift perturbation calculations

Weighted chemical shift perturbations (CSPs) were calculated using the formula below:

$$CSP = \sqrt{(\Delta\delta_C / 11.8)^2 + (\Delta\delta_H)^2}$$

where $\Delta\delta_C$ and $\Delta\delta_H$ are the differences in ^{13}C and ^{1}H chemical shifts, respectively. The weighing factor that accounts for dispersion was determined by selecting the Cα range (10.5 ppm) and the Hα range (1.4 ppm).

2d. Relative abundance analysis of dipolar-based 2D ^{1}H-^{13}C correlation spectra

By assuming that the contribution for every CH pair of a polysaccharide species in the spectrum is equivalent we can determine a rough estimate of the relative contribution per polysaccharide species. The peak volumes (from 700 MHz data) resulting from the integration areas shown in Fig. 2 are reported in Table S6. By determining the total volume of all polysaccharide peaks, relative contributions per peak can be determined. In Table S7 the percentual contribution of specific polysaccharides to all rigid cell wall polysaccharides is calculated by multiplication of the relative contribution by the number of expected CH resonances in the ^{1}H-^{13}C 2D correlation spectrum. To solve the α-(1,3)-glucan polysaccharide contribution we used integral #4 and the average contributions calculated in Table S6. For this we assumed integral 4 could be simplified to: Integral 4 = Ch + A + B.

2e. Relative abundance analysis of dipolar-based Double Quantum Single Quantum ^{13}C-^{13}C 2D spectra

The relative abundance of polysaccharide species as well as data error was determined by following a method published by Chakraborty *et al*.[16]. Peak volumes (see Table S8 for source data) and relative monomer abundance (see Table S9) were determined using the integration regions shown in Fig. S3.

2f. Relative abundance analysis of scalar-based ^{1}H-^{13}C 2D correlation spectra

By assuming that the contribution for every CH pair of a polysaccharide species in the spectrum is equivalent we can determine a rough estimate of the relative contribution of polysaccharide species in scalar-based experiments by focusing solely on the carbohydrate C1 region (here defined in the ^{13}C dimension as between 90 ppm and 107 ppm). Due to sharp linewidth, peak height (from 700 MHz data) was used to determine relative contributions per polysaccharide. Source data is found in Table S10 and Table S11 displays a summary of the percentual contribution of specific polysaccharides to all flexible cell wall polysaccharides.

References

[1] F. H. J. Schuren, J. G. H. Wessels, *Gene* **1990**, *90*, 199-205.

[2] M. van Eijk, S. Boerefijn, L. Cen, M. Rosa, M. J. H. Morren, C. K. van der Ent, B. Kraak, J. Dijksterhuis, I. D. Valdes, H. P. Haagsman, H. de Cock, *Med Mycol* **2020**, *58*, 1073-1084.

[3] H. L. Ehren, F. V. W. Appels, K. Houben, M. A. M. Renault, H. A. B. Wosten, M. Baldus, *Cell Surf* **2020**, *6*, 100046.

[4] S. Puanglek, S. Kimura, Y. Enomoto-Rogers, T. Kabe, M. Yoshida, M. Wada, T. Iwata, *Sci Rep* **2016**, *6*, 30479.

[5] T. Fontaine, C. Simenel, G. Dubreucq, O. Adam, M. Delepierre, J. Lemoine, C. E. Vorgias, M. Diaquin, J. P. Latge, *J Biol Chem* **2000**, *275*, 27594-27607.

[6] E. M. Dahmane, Taourirte, M., Eladlani, N., Rhazi, M., *J. Polym. Anal. Charact.* **2014**, *19*, 342-351.

[7] J. B. Bouwstra, Kerékgyárto, J., Kamerling, J. P., Vliegenthart, J. F. G., *Carbohydr. Res.* **1989**, *186*, 39-49.

[8] M. I. Bilan, A. A. Grachev, A. S. Shashkov, M. Kelly, C. J. Sanderson, N. E. Nifantiev, A. I. Usov, *Carbohydr Res* **2010**, *345*, 2038-2047.

[9] X. Kang, W. Zhao, M. C. Dickwella Widanage, A. Kirui, U. Ozdenvar, T. Wang, *Journal of Biomolecular NMR* **2020**, *74*, 239-245.

[10] F. R. Smiderle, L. M. Olsen, E. R. Carbonero, C. H. Baggio, C. S. Freitas, R. Marcon, A. R. Santos, P. A. Gorin, M. Iacomini, *Eur J Pharmacol* **2008**, *597*, 86-91.

[11] G. J. Gerwig, Van Kuik, J. A., Leeflang, B. R., Kamerling, J. P., Vliegenthart, J. F. G., Karr, C. D., Jarroll, E. L., *Glycobiology* **2002**, *12*, 499-505.

[12] E. L. Ulrich, H. Akutsu, J. F. Doreleijers, Y. Harano, Y. E. Ioannidis, J. Lin, M. Livny, S. Mading, D. Maziuk, Z. Miller, E. Nakatani, C. F. Schulte, D. E. Tolmie, R. Kent Wenger, H. Yao, J. L. Markley, *Nucleic Acids Res* **2008**, *36*, D402-408.

[13] Y. Wang, O. Jardetzky, *Protein Sci* **2002**, *11*, 852-861.

[14] W. Lee, M. Tonelli, J. L. Markley, *Bioinformatics* **2015**, *31*, 1325-1327.

[15] S. Luca, D. V. Filippov, J. H. van Boom, H. Oschkinat, H. J. M. de Groot, M. Baldus, *J Biomol NMR* **2001**, *20*, 325-331.

[16] A. Chakraborty, L. D. Fernando, W. Fang, M. C. Dickwella Widanage, P. Wei, C. Jin, T. Fontaine, J. P. Latge, T. Wang, *Nat Commun* **2021**, *12*, 6346.

Author contributions

AS, SB, and MB designed and AS, SB and DB recorded the ssNMR experiments. FK, JvN and MT prepared ssNMR samples. AS performed the ssNMR data analysis. EV and HdC determined inhibition of growth by CATH-2 of *S. commune*. AS, HdC, HW and MB wrote the manuscript. All authors reviewed it and agreed to its publication.
